# On Digitalis and Its Uses

**Published:** 1866-12

**Authors:** Jos. Adolphus

**Affiliations:** Hastings, Michigan


*On Digitalis and its Uses.* JOS. ADOLPHUS, M. D., of Hastings, Michigan.

While the profession is in serious quest after new remedies, we have been sadly at fault in neglecting old and valuable ones, in consequence of not having sufficiently attended to their therapeutic and physiological operation. Such is the case with the article now under consideration. It is not unusual to find digitalis stated in the books as an arterial sedative, whose operation is particularly on the action of the heart. In part this is true, but the true usefulness of digitalis is not to be found in its arterial sedative qualities. I will call attention to the use of this article in general practice, and then a more correct judgment may be formed of its usefulness.

A woman æt. 38; anemic, with a small, weak, frequent pulse, 115 per minute; hearts action weak, with deficient aortic valves; general œdema; pupils contracted in the morning, and much dilated at evening; nervous system quite irritable; tongue moist, flabby, broad, pale, and tremulous when protruded; respiration short, 23 per minute; occasionally skin colored with a jaundice hue; complains frequently of headache; feet and hands most genererally cold and clammy, and for a week back complains of insomnia. When I first saw her she had a hacking cough, anorexia, feeble pulse, and sleeplessness; she was attended by a neighboring physician, who called her case tubercu losis. I immediately put her under the tincture digitalis, according to the U. S. P., gtt. x. three times a day; no other remedy. Five days after, the pulse was 85 per minute, and strong; skin moist and warm, and œdema disappearing; color of skin more natural; respiration reduced to 18 per minute; relish for food returning, and sleep better, being more refreshed; heart's action more regular. At the commencement of the second week, all the symptoms quite improved. She then took cod liver oil, and thirty-six days after this treatment was begun, she was well enough to leave off the digitalis which she had taken in four-drop doses after the first eight days. She continued the oil for two months longer, and has from that date (seven months) onward had no return of the bad symptoms, though the valvular disease is still present.

*Commentary.* This case is one of four, all treated in like manner, and shows that digitalis is a tonic to the circulating system.

No unusual flow of urine occurred during the disappearance of the œdema, and with its disappearance also went the jaundiced discoloration of the skin. The restored cardiac force was followed by improved digestion and nutrition; the brain was supplied with superior life-force through the improvement in the blood. That the power of the remedy was in fact directed toward the nutritative and nervous system cannot be denied, inasmuch as the excito-secretory system of nerves were renewed in life-force.

The next class is that of pneumonia. Seven cases of pneumonia, treated with digitalis tinctured.

*Case* 1. Boy eleven years old; full habit and strong physical developments; was attacked with single pneumonia of right lung; condition of patient eighteen hours after first chill was as follows: Pulse 105, sharp, full; skin hot; face flushed; cough frequent and dry; urine scanty and high-colored; headache; eyes suffused and watery; pupils sensitive to light; respiration quick and short, painful; percussion dull up to the sixth rib; respiration higher up, purile; over the inflamed part the sound was mixed with crepitant rale, dry rhoncus, etc., etc. Commenced the treatment with two drops of tincture of digitalis, every hour; enveloped the chest in a mush poultice. Ten hours after, the cough was not so urgent, and the pulse was lowered five beats. Thirty-six hours later fine crepitation was heard; puerile respiration not so strong; urine more copious. In forty-eight hours more resolution was effected.

*Case* 2. Man, 86 years of age; bilious habit, mixed with nervous; attacked with chills and rigors, followed by high fever; cough; painful respiration, and headache. I saw the case sixty hours after the first chill. Ausculation and percussion revealed confirmed pneumonia. Pulse 100; tongue sharp, red at tips and edge, base dirty white; pappillæ prominent, especially toward lower third. Skin hot and pungent; breath offensive; eyes red and suffused; pulse soft and quick, quite easily compressed and lost under the finger; lower half of left lung involved; cough very harassing; sputa thick and tough; urine scanty, ammoniacal and high colored.

Commenced the treatment with ten drops of tincture of digitalis every four hours, until six doses are taken, and then five drops every four hours. Sixty hours after, symptoms began to yield. Sputa began to grow less tenacious. Pulse stronger, and not so full nor so soft. Fifth day of treatment the crepitant fine sound began to be heard. On ninth day patient considered convalescent.

*Case* 3. Woman, 62 years old; of broken down constitution; was attacked with pneumonia in both lungs, but severest in right. After the second stage was well established, (which was on the fifth day) I saw her. Pulse 130, quite small and weak; dullnes all over lower half of right lung, and one-fourth of left; no respiratory murmur or sound of any kind; tongue dry, covered with dark-brown fur; respiratory movements quick and short. Case esteemed of a typhoid type.

Commenced treatment with three drops of tincture of digitalis, every three hours. In eighty-four hours the pulse grew stronger and firmer; and in sixty hours more, respiration began to be heard in the upper edge of the left lung, and so on gradually, and by the eighteenth day respiration was restored all through both lungs.

These three cases are types of the whole, but let us read a lesson.

The great power of the remedy was to strengthen the heart's action, for the pulse was more or less strengthened before forty-eight hours of treatment. Nutritive life was developed anew, for the life-forces seemed to be called into renewed action.

In all these cases quinine was used, and mush jackets applied to the whole chest—which, by-the-by, is one of those great adjuncts to the successful treatment of all lung complaints, which we must never neglect.

